# THE IMPACT OF AN EARLY-STAGE DYADIC CARE PLANNING INTERVENTION ON CAREGIVER BURDEN

**DOI:** 10.1093/geroni/igz038.797

**Published:** 2019-11-08

**Authors:** Silvia Orsulic-Jeras, Carol Whitlatch, Justin Johnson

**Affiliations:** Benjamin Rose Institute on Aging, Cleveland, Ohio, United States

## Abstract

Evidence supports the development of proactive, dyadic interventions for used in early-stage dementia. This type of intervention leads to more effective decision making which can reduce subsequent caregiver stress and burden. SHARE (Support, Health, Activities, Resources, Education), a six-session counseling-based intervention, encourages and supports care dyads to have important discussions about health care preferences that are often delayed or avoided until later-stage dementia. Typically, both the PWD and caregiver assume that most of the help will be delivered by the caregiver. SHARE aims to expand the network of care by evenly distributing care task responsibilities from the caregiver alone to other potential sources of support: family/friends and paid service providers. Early-stage dyads (n=63) successfully created a balanced and manageable plan of care with the help of their SHARE Counselor over a 6-month period. Follow-up interviews assessed care dyads from 1.5 to 2 years after their participation in SHARE ended. Analyses determined change in PWD ADL needs from enrollment (T1) to two years later (T2) and impact on caregiver task burden. Paired samples t-tests indicated a significant increase in PWD need for assistance with 18 ADLs, from a T1 mean of 8.76 to a T2 mean of 11.34 (t=-6.72, p=.000). This paper examines: 1) the benefits and challenges of creating a Care Plan in the early stages of dementia, 2) how SHARE dyads utilized their Care Plans when PWDs needed assistance, and 3) if reliance on family/friend and paid service provider options were realistic over time.

